# Ultrafiltration as an alternative purification method for bovine serum albumin-fluorescein isothiocyanate bioconjugate

**DOI:** 10.17912/micropub.biology.001731

**Published:** 2025-07-23

**Authors:** Catherine A. Jackson, Elise L. McKean, John M. Hawdon

**Affiliations:** 1 Microbiology, Immunology, and Tropical Medicine, George Washington University, Washington, Washington, D.C., United States; 2 Biological Sciences, George Washington University, Washington, Washington, D.C., United States

## Abstract

The bioconjugation of proteins with fluorescent probes is a popular method for protein visualization and has widespread applications across scientific fields. This study assessed ultrafiltration as an alternative purification method following the bioconjugation reaction that tags fluorescein isothiocyanate (FITC) to bovine serum albumin (BSA). Success of the purification was determined by (1) UV-Vis spectroscopy, comparing our sample to a commercially available product, and (2) use in an in vitro bioassay. Centrifugal ultrafiltration devices with a molecular weight cut off below the molecular weight of the conjugated protein were successful in retaining the purified product and clearing unreacted FITC from solution.

**Figure 1. UV-Vis spectrum and in vitro functionality of FITC-BSA using different purification methods.**
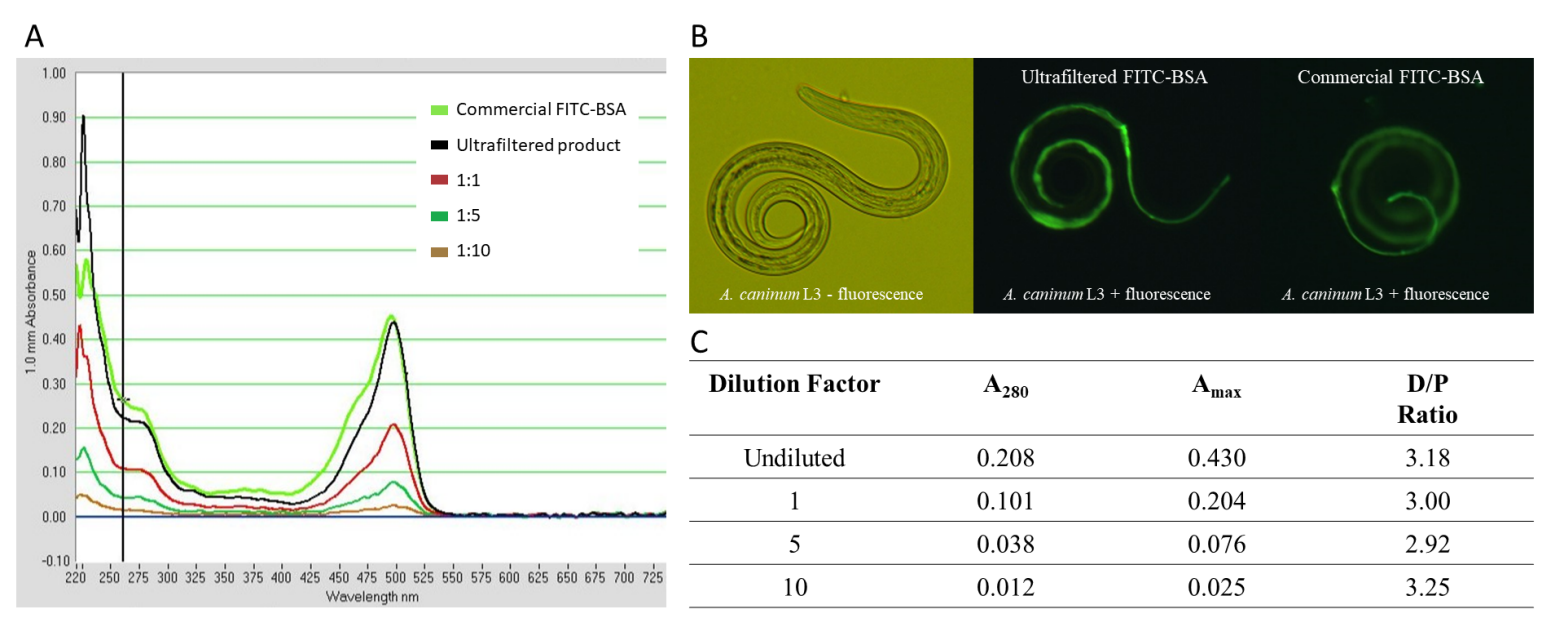
(A) UV-Vis spectrum obtained with a Nanodrop-1000 comparing the absorbance of commercially obtained FITC-BSA and various dilutions of our ultrafiltered sample. (B) Confirmation of FITC-BSA fluorescence in vitro for the commercial product (rightmost panel) as well as our product following ultrafiltration (middle panel). (C) Dye/protein ratios calculated using the absorbance values obtained at 280 nm and 495 nm for multiple dilutions of the ultrafiltered product.

## Description

Fluorescein isothiocyanate-conjugated bovine serum albumin (FITC-BSA) is a fluorescently tagged protein with a variety of biochemical uses spanning numerous scientific disciplines. Serum albumins are generally popular due to their low cost and stability (Barbero et al. 2009), and conjugation to a fluorescent probe greatly facilitates protein visualization. Protein localization is among the most common uses for FITC-BSA (Pietra & Johns 1996), as the fluorescent tag enables visualization of cellular transport, dynamics, and kinetics (Chaikhumwang et al. 2020, Lichtscheidl et al. 2021, Lommen et al. 2021). An example of one of its unique uses arises from the field of parasitology, where FITC-BSA is used in larval feeding activation assays with the hookworm species *Ancylostoma caninum* and other species to determine the feeding status of larvae under various conditions (Hawdon & Schad 1990, Wang et al. 2009, Huang et al. 2010). In this assay, infective stage larvae are incubated in host-like conditions that stimulate a feeding response. To determine whether the worms have been stimulated to feed, FITC-BSA is added near the end of the incubation period. Feeding larvae will ingest the fluorescently tagged protein, which can be visualized by fluorescent microscopy. One of the major benefits of using FITC-BSA in this assay, as opposed to free FITC, is the solubility change that occurs after bioconjugation. On its own, FITC is sparingly soluble in aqueous buffers, and can only be dissolved in ethanol, dimethyl sulfoxide (DMSO), or dimethylformamide (DMF). Highly polar solvents such as these are generally not considered amenable for in vitro assays with live nematodes. DMSO, even in small amounts, has been observed to have negative impacts on nematode development (Mickiewicz et al. 2025), and ethanol is known to be a highly effective surface disinfectant against hookworm larvae (Kines et al. 2021). Conversely, BSA tagged with FITC is soluble in aqueous solutions up to 10 mg/mL, circumventing a major confounding variable concerning in vitro larval assays.

FITC-BSA is commercially available through Sigma-Aldrich, though some researchers may prefer to prepare it themselves. This is achieved through bioconjugation, in which one molecule becomes covalently linked to a protein, carbohydrate, or nucleic acid (Hermanson 2008). In the bioconjugation reaction that yields FITC-BSA, the electrophilic carbon on the isothiocyanate group of the fluorophore molecule attacks the nucleophilic N-terminal amine on the BSA protein (Jorbágy & Király 1966). A stable covalent bond is formed, and this protein may now be visualized using fluorescence.

Several complications may arise when performing this tagging reaction, chief among them being access to purification methods in laboratories that lack the resources to perform gel filtration or advanced dialysis techniques. The tagging reaction itself is rather simple and can be performed with a few basic chemicals that would be easily accessible to most researchers. However, the purification of the FITC-BSA product may present a major impediment for some labs. In this paper, we report an alternative purification method following BSA bioconjugation with FITC.

In this study, we were able to reliably purify a FITC-BSA product, removing unreacted free FITC, using a centrifugal filter device with a molecular weight cutoff (MWCO) of 30,000 Daltons (Da). This filter size allowed any FITC (389.3 Da) that was unbound to albumin (66.5 kDa) to be pushed through the membrane into the filtrate. This left only FITC-BSA and unbound BSA in the retentate, which could be resuspended and diluted appropriately in any aqueous buffer, ready for use.

One obvious caveat is that excess albumin is not removed, leaving unreacted protein in the final product. However, since the tagging reaction is performed in a significant excess of FITC, the amount of unconjugated BSA remaining is likely negligible, provided that the reaction has been successful. To produce a qualitative and quantitative assessment of the success of purification, we employed UV-Vis spectroscopy and our larval feeding assay. We first compared an aliquot of our filtered product to a known standard (lyophilized FITC-BSA purchased from Sigma Aldrich resuspended in PBS). The UV-Vis spectrum obtained showed that the absorbance profile of our product was nearly identical to that of the commercially obtained sample ( Figure 1A ). Next, we used our ultrafiltered product in the in vitro larval activation assay. After the appropriate incubation period, activated L3 were successfully labeled with FITC-BSA, as shown by fluorescent microscopy ( Figure 1B ). As a final test, we made several dilutions of the product to ensure that wavelengths at which absorption peaked remained consistent. Using the absorption values at 280 and 495 nm, the dye-to-protein (D/P) ratio was calculated for each dilution. The value fell within 2.9-3.2 for each sample, indicating an average of 3 molecules of dye per molecule of protein ( Figure 1C ). This D/P ratio reflects a successful tagging procedure, as a previous study investigating another type of fluorescent tag found that BSA utilizes up to five binding sites (Togashi & Ryder 2008) and fluorescent probe interactions only occur at labelling ratios greater than 3:1 (FITC:BSA) (Hungerford et al. 2009). Based on the spectral similarity to commercial FITC-BSA, the consistently reasonable D/P ratios, and the success in the activation assay, we concluded that purification by ultrafiltration was effective.

While dialysis and gel filtration remain an excellent method of purification for fluorescently tagged proteins, we have shown that ultrafiltration is possible if the former options are not feasible. FITC-BSA represents a convenient and affordable option for researchers in a variety of fields. Having alternative purification options for tagging procedures increases scientific accessibility and may provide a necessary avenue for researchers lacking other options.

## Methods

*Bioconjugation of BSA with FITC*

The tagging reaction was performed using an established protocol (Barbero et al., 2016). In brief, a 2 mg/mL solution of BSA in 0.1 M sodium carbonate (pH 9) was labelled using a 1 mg/mL solution of FITC dissolved in anhydrous DMSO. The reaction was left to proceed in the dark at 4C overnight (approximately 16 hours). The reaction was quenched with ammonium chloride to a final concentration of 50 mM and left for another 2 hours. Purification was performed immediately after quenching.

*Purification of FITC-BSA using Ultrafiltration Tubes*

In accordance with the specific molecular weights of the reactants described, a 15 mL Amicon® Ultra centrifugal filter device with a MWCO of 30 kDa was utilized. Prior to filtration, the centrifugal filter membrane was conditioned with 2 mL of phosphate buffered saline (PBS) and spun at 3000 x g for 2 minutes, after which the filtered PBS was discarded. The bioconjugation of FITC to BSA was performed in a 15 mL conical tube with 11 total mL of solution. At the end of the reaction, the entire solution was transferred and split between 2 ultrafiltration tubes containing 5.5 mL each. The filter tubes were centrifuged at 3000 x g for 15 minutes, after which approximately 500 µL of retentate remained. The filtrate, containing the free FITC, was discarded. Two washes of the retentate were performed at the same speed after the addition of 10 mL of PBS to the upper chamber. Each successive wash required less time to filter as the retentate continued to lose excess reagent. Thus, three total centrifugations were required: the first for 15 minutes, the second for 10 minutes, and the third for 5 minutes. It is important to monitor the volume of liquid that remains in the upper chamber of the tube after each centrifugation to ensure that the retentate does not become too dry. After three washes, the retentate was resuspended in 10 mL of PBS and transferred to a 15 mL conical tube. At this point, the solution may either be used as it is or diluted further with PBS. The resulting solution may be stored at 4°C for short term use, or -20°C for long term storage.

*Determination of D/P via UV-Vis Spectroscopy*

Immediately following purification and dilution, the absorbance of the final product was measured at 280 nm and 495 nm using a Nanodrop-1000. The formula used to calculate the degree of labelling is shown below:

D/P = (A _max_ * ɛ _prot_ ) / (ɛ _dye_ *(A _280_ -c*A _max_ ))

Where A _280_ and A _495_ are the absorbance values at 280 nm and 495 nm, respectively. ɛ _prot_ and ɛ _dye_ are the respective coefficients of extinction of BSA (43824 M ^-1^ cm ^-1^ ) at 280 nm and FITC (75000 M ^-1^ cm ^-1^ ) at 495 nm, as obtained from the literature (Pace et al. 1995, Manczyk et al. 2017). C is a correction factor needed to account for the absorbance at A _280_ that is contributed by the fluorophore, which is equal to 0.3 for FITC.

*Confirmation of Function via Parasite Assay*

To verify that our FITC-BSA product retained its desired function, it was used in our larval activation assay. *A. caninum* L3 were incubated in host-like conditions for 24 hours as previously described (Hawdon & Schad 1990, 1991, 1993). Two hours before the end of the incubation period, 100 µL of FITC-BSA was added to wells. At 24 hours incubation, L3 were washed in PBS and examined with fluorescent microscopy using a fluorescein filter cube. Success of the conjugated protein function was determined by visualization of fluorescence in the intestinal lumen of the L3.

## Reagents

**Table:** 

Reagent	Details	Vendor
Anhydrous dimethyl sulfoxide (DMSO)	CAS No. 67-68-5, 100 mL	Sigma Aldrich (Burlington, MA)
Gibco™ PBS	Catalog No. 10-010-023 500 mL, pH 7.4	FisherScientific (Boston, MA)
Fluorescein 5(6)-isothiocyanate	CAS No. 27072-45-3, 100 mg	Sigma Aldrich (Burlington, MA)
Bovine serum albumin	CAS No. 9048-46-8, 10 g	Sigma Aldrich (Burlington, MA)
Sodium carbonate anhydrous (powder)	CAS No. 497-19-8, 500 g	FisherScientific (Boston, MA)
Ammonium chloride	CAS No. 12125-02-9, 500 g	ICN Biomedicals (Aurora, OH)
Albumin-fluorescein isothiocyanate conjugate	Catalog No. A9771, 250 mg	Sigma Aldrich (Burlington, MA)
Amicon™ Ultra-15 centrifugal filter units	Catalog No. UFC903024 30 kDa MWCO	MilliporeSigma (Bedford, MA)
